# Supplementary material for: Experiences of psychotropic medication use and decision-making for adults with intellectual disability: a multistakeholder qualitative study in the UK
Source: BMJ Open. 2019 Nov 27;9(11):e032861. doi: 10.1136/bmjopen-2019-032861 (PMC6887070; doi:10.1136/bmjopen-2019-032861)
Supplement: Supplementary data [file bmjopen-2019-032861supp001.pdf]

Topic guide – people with ID

To be supplemented with visual information and prompts

- What do you think about the medication you take for mental health?

- Do you talk about psychotropic medication with the psychiatrist?
  - What has this been like?
  - Do they ask what has been good about taking medication?
  - Do they ask what has been bad about medication?

- Who is involved in decisions about psychotropic medication?
  - Do you want to be involved?
  - Are you involved?
    - If not, why?
  - Is anyone else involved (e.g. carer, family member)?
    - How are they involved?
    - What do you think about them being involved?

- Do you feel that you have a choice about medication?
  - Does the psychiatrist ask you what you want to do with medication?
  - Have they listened to your views?

- What if you were worried about your medication?
  - What if you had a problem with your medication?

- What should the doctor think about when they are prescribing medication for you?
  - What is important to you?
  - What do you want to know about the medicine?

- What would make it easier to talk to the doctor about medication?

Topic guide – family carers

- What has been your experience when psychotropic medication has been prescribed for your relative?

- Who is involved in decisions about psychotropic medication?
    - How is your relative involved in the decision?
    - Are you involved?
    - Who else is involved?
    - Is/was your level of involvement what you would like?

- Is medication reviewed after it has been prescribed?
    - How?
    - What was the review like?
    - Are you involved in this?
    - Is the review effective?

- How were/are decisions to continue, stop, or change medication made?
    - Have you and your relative been given a choice about medication?

- Do you discuss medication with the psychiatrist at appointments?
    - Do you think that you know enough about the medications?
    - How would you know if medication is working or not working?
    - Do you have a method for recording the positive and negative effects of medication (e.g. rating scales)?
    - What if there is a problem with medication?

- What should be thought about when medication is reviewed?

- What might make it easier for you or your relative to give your views about medication?

Topic guide – paid carers

- What has been your experience when psychotropic medication has been prescribed for the people you support?

- Who is involved in decisions about psychotropic medication?
    - How is the person you support involved in the decision?
    - Are you involved?
    - Should you be involved?

- Is medication reviewed after it has been prescribed?
    - How?
    - What happens in the review?
    - Are you involved in this?
    - Is the review effective?

- Who makes decisions to continue, stop, or change medication?
    - How are these decisions made?
    - Have you and the person you support been given a choice about medication?

- Do you discuss medication with the psychiatrist at appointments?
    - How able do you feel to contribute to this discussion?
    - Do you think that you know enough about the medications?
    - How would you know if medication is working or not working?
    - Do you have a method for recording the positive and negative effects of medication (e.g. rating scales)?
    - What if there is a problem with medication?

- What should be thought about when medication is reviewed?

- What might make it easier for you or the person you support to give your views about medication?
